# Supplementary material for: Sustainability of an evidence-based intervention supporting transition to independent care for youth living with HIV in Kenya
Source: PLOS Glob Public Health. 2025 Jan 13;5(1):e0004111. doi: 10.1371/journal.pgph.0004111 (PMC11981535; doi:10.1371/journal.pgph.0004111)
Supplement: S1 File — (DOCX) [file pgph.0004111.s002.docx]

| **ATTACH Study – AHISA Supplement** |
| --- |
| **Healthcare Worker**  **Individual Interview Discussion Guide** |

**Facilitator Instructions:** Before beginning the interview, make sure each healthcare provider has completed the informed consent process and provided their demographic information using the short demographic survey.

Note the following questions provide a guide for the interview. Try to ask all the questions below in the order given, but it is more important to maintain the flow of discussion. Suggested probes have been included.

******************************************************************************************************************************

**Welcome and Introductions**

**Before turning on the recorder, start with the following introductory script:**

*Hi, my name is ____________ and this is (introduce note-taker). Thank you for agreeing to participate in the focus group discussion today.*

*As a healthcare provider at this clinic, you are offering the Adolescent Transition Package to adolescents and young adults. This is a set of tools that helps facilitate disclosure of HIV status to younger adolescents, and transition to adult care for older adolescents and young adults. This is a newly developed set of tools that was implemented as part of a clinical trial called the ATTACH study. Your clinic either used the tools during the study as one of the intervention sites, or was trained to use the tools at the end of the study. Your clinic is one of the few clinics that has ever used these tools, so we want to hear from you about your experience using the Adolescent Transition Package materials at your clinic.*

*We are interested in all your thoughts and ideas about the intervention tools, especially barriers and facilitators to implementing the intervention tools at your facility. During our discussion today, I will ask you questions that you are free to answer in any way you wish. Feel free to elaborate on any of your points. If a question is unclear to you, please feel free to ask me to explain it.*

*I would like to record the discussion, so I don’t miss anything that you say. I will not include your full name on any documents or in the recording. Your responses will be kept confidential, which means we will keep what you say private from others. Is it okay if I record our discussion?* [Wait for the participant to give verbal consent to recording]

*Before we start, I would like to remind you that there are no wrong answers during our discussion. We are interested in knowing what you think, so please feel free to be open and share your point of view. We hope you can help us understand how to best adapt these intervention tools for use in other clinics in the future. Your comments about what didn’t work are just as helpful as your comments about what did work.*

*If you want to stop the discussion at any time, just let me know. Do you have any questions for me before we get started?* [Wait for participants to respond – answer any questions they have]

*I am turning on the recorder now.*

****************************************************************************************************************************

**Before beginning interview questions, please read the following script for the recording:**

*Today is [day of week], [month, day, year] and it is now [time of day]. This is interview [ID number] conducted at [discussion location].*

**You are now ready to begin asking the questions outlined below.**

*****************************************************************************************************************************

**Semi-Structured Interview Discussion Questions**

1. *First, can you tell me a little about your clinic/facility, including how things work now, and what the overall climate [climate = energy, feel, environment] is like?*

**[Note: Context/Environment - This question asks about the clinic generally, and the overall environment and structure at the clinic. It is not specifically asking anything about the Adolescent Transition Package at this point]** (CFIR domains: Inner setting and Individuals involved in the implementation)

- - ***Possible probes:*** relationships with supervisors and colleagues, communication structures and processes in the clinic, relationships with others in the clinic, relationships between this clinic and other clinics/facility, perceptions/beliefs on the ability to try new services/processes in the clinic (both individually and collectively as a team), willingness to try new things or change clinic processes (both individually and collectively as a team), changes since the ATTACH trial

1. *When you think back to when you first learned about the ATP, what did you think of the tools and procedures that are part of this package?*

**[Note: This question asks about initial/early implementation, including training and then initial perceptions during the first month or two after being trained]** (CFIR domain: Intervention Characteristics, Characteristics of Individuals)

- - ***Possible probes:*** training delivery and design, initial beliefs about the intervention, overall processes of delivering new intervention services, comparison to previously offered services and processes for offering those services in the clinic, initial thoughts on challenges or benefits of using the ATP, whether they are acceptable (you like and welcome the transition and disclosure tools) and feasible (seems possible or doable to use in your clinic)

1. *What is it like now to use the ATP tools? What has changed, and why do you think it’s different?*

**[Note: This question asks about implementation of the ATP currently, ~12 months post trial]** (CFIR domain: Intervention Characteristics, Process of Implementation)

- - ***Possible probes:*** fidelity (fidelity = implementation as trained) to the study design/planned implementation, beliefs about the intervention, comparison to previously offered services, challenging and positive experiences with using the ATP, suggestions for improving fidelity (use of the booklets, tracking tools, and readiness assessments), acceptability (making it something you like/welcome), and feasibility (use of ATP tools seems possible to do)

1. [*I’d like to learn more about other factors, such as people in the community or external partners supporting HIV care at this clinic, and external factors such as policies that may impact implementation of the ATP] Who has been supportive of implementing the ATP? What has helped keeping the momentum after the RCT? What could have been done better?*

**[Note: This question asks about WHO has had a key role in helping with implementation of the ATP in the clinics; some providers may be less aware of buy in from community and country/county leaders]** (CFIR domain: Outer Setting)

- - ***Possible probes:*** Support during ATTACH trial and role of the study team, support post-trial, overall distribution of responsibilities within the clinic/facility, leaders within the clinic or facility, influence or buy in from community, county leaders, country leaders, new guidelines or policies

1. *What should we be thinking about if we wanted to integrate the ATP into routine care in other facilities/clinics in Kenya?*

**[Note: This question asks about the future of scaling up the ATP to other health facilities in Kenya]** (CFIR domain: Process of Implementation, Intervention Characteristics, Inner Setting, Outer Setting)

- - ***Possible probes:*** Beliefs about intervention effectiveness and impact (eg: do the booklets facilitate improved disclosure or transition, are more AYA retained in care, are AYA better with adherence or do they have improved viral suppression), beliefs about what would need to be adapted for even longer term sustainability, beliefs about what would need to happen for scalability (expansion of the ATP to other clinics and settings), suggestions for who would need to be involved in scaling to other settings (MOH, clinic leadership, etc.), prioritization (which counties to start with, which clinic)

*As we finish talking today, are there any other questions we should be asking people like you to help us understand whether and how the ATP can be implemented at your facility/clinic?*

*Is there anything about the implementing the ATP, or just about the transition process for AYA, that you think is important to tell me, but I didn’t ask about?*

*These are all of the questions I have for you today. Thank you for your time. If you do not have any further questions or comments, I will now turn off the recorder.*

******************************************************************************************************************************
