# Supplementary material for: Sustainability of an evidence-based intervention supporting transition to independent care for youth living with HIV in Kenya
Source: PLOS Glob Public Health. 2025 Jan 13;5(1):e0004111. doi: 10.1371/journal.pgph.0004111 (PMC11981535; doi:10.1371/journal.pgph.0004111)
Supplement: Attachment — Submitted filename: Response to reviewers.docx [file pgph.0004111.s003.docx]

July 22nd, 2024

Dear Editor,

We are grateful for the thoughtful comments from the reviewers and editorial team on our manuscript: “Sustainability of an evidence-based intervention supporting transition to independent care for youth living with HIV in Kenya.”

We have responded to each comment in the text below and revised the manuscript accordingly. We also included a complete copy of PLOS’ questionnaire on inclusivity in global research.

We appreciate your consideration of this paper for publication. Please contact us if you have any further questions or require additional information regarding this submission.

Sincerely,

Alina Metje, MD, MPH

Research Associate

Department of Global Health

University of Washington, USA

**Reviewer #1**

The area of study is of high importance in contemporary public health program interventions. The study was subjected to rigorous qualitative methods adopting the Consolidated Framework for Implementation Research. Data analysis (content analysis) using ATLASti was scientifically sound and results were profound and generalizable.

*Thank you very much for this comment. We appreciate your review of this paper.*

**Reviewer #2:**

A. On the Scope:

1. Is that transition of Adolescent youth HIV services to independent care or sustainability of transition of Adolescent youth HIV services to independent care?

*This analysis focused on understanding the continued implementation (sustainability) of the Adolescent Transition Package (ATP) intervention which prepares adolescents living with HIV to transition from pediatric/youth specific services to independent care.* *We have revised the introduction section to specify this.*

2. When do we conduct qualitative study and how did you taken this issue in to account while doing this research?

*This study is a follow-on study to the randomized clinical trial. This study aims to evaluate determinants of sustainment and fidelity post trial. The qualitative work provides a deeper dive into the experiences and perspectives of the HCWs delivering the intervention (the ATP) to understand the contextual factors that influence sustained implementation and fidelity after the trial ended. We have included this sentence in the introduction section: “Using a qualitative approach to evaluate these outcomes provides a deeper understanding of HCW’s perspectives and experiences with implementation, which can inform approaches to facilitate scale-up and long-term integration into routine health systems.”*

3. Adolescence is the age of transition hence the specific selection of adolescents with sensitive illness like HIV and the decision capacity is questionable?

*We agree that adolescents are in a transitional phase and vulnerable, particularly YLH. This analysis consisted only of health providers who delivered the intervention to adolescents, not the adolescents themselves.*

4. Why don’t you conduct among other segment of the population E.g., adults?

*As highlighted in Q3, we clarify that this analysis was only focused on the perspectives of health providers (all adults) providing services to youth.*

5. What is the difference and similarity of independent care with self-management practice?

*Independent care and self-management are similar concepts focused on self-reliance and personal responsibility, and both reflect the experience of needing to manage one’s own care. In our manuscript, we consistently chose the term independent care to discuss this concept.*

6. Why sustainability or continuity after post trial?

*This study is within the field of implementation science, where the goal is to help move effective interventions to larger scale implementation, including sustained use in clinical settings. Within this field of research, it is important to identify factors that lead to sustained use beyond the RCT period to provide information for future implementation and continued evaluation of intervention impact on health outcomes.*

7. Is that post trial evaluation or sustainability assessment?

*The effectiveness of the ATP was previously evaluated in a cRCT. This analysis is an assessment of the sustainability of intervention implementation after the original trial ended. Information on the original trial and goal of this specific project are included in the introduction and methods sections of the manuscript.*

8. How do you assess sustainability in the absence of the beneficiaries meaning the adolescents?

*In implementation science, sustainability refers to continued implementation of an intervention over time. For this analysis, sustainability was defined as the continued implementation of the Adolescent Transition Package in health facilities. We were interested in what made it harder or easier for HCWs, to deliver the tool after the the trial ended. We plan to look at the sustainability of long term treatment outcomes at the adolescent level in future work.*

B. On the Background and methods

1. The background should establish strong rationale for the ‘sustainability’ ‘transition’ of the care and the appropriateness of the framework and its advantage over others?

*We have included details in the introduction on our engagement with the Kenya National HIV program, who have expressed interest in scaling the ATP beyond the RCT. An understanding of post trial sustainment provides the program with data that will be crucial in future intervention scale-up.*

2. The specific type of the study design is not mentioned

*We have included details of the study design in the methods section*. This section reads:

“*We conducted a qualitative evaluation involving semi-structured individual interviews with HCWs one year after the original ATTACH trial ended*…*We conducted a directed content analysis to identify key determinants of ATP sustainment and fidelity.”*

3. The use of software for analysis is not described

*As noted in the methods section, this analysis used ATLAS.ti version 23. We have added additional details describing the software features to the methods section, which now reads:*

*“ATLAS.ti 23, a qualitative data analysis software providing advanced tools for coding, visualizing, and interpreting qualitative data, was used for data management and analysis.”*

4. Data is not collected from the adolescents and other segments of the population

*The focus of this analysis was on the perspectives and experiences of health care workers delivering the ATP as they provide unique and invaluable feedback on how best to facilitate long term delivery of this intervention. We agree that the experiences of adolescents and other members of the community are also important in understanding long term implementation of this intervention and look forward to exploring this in more detail in future work.*

5. Trustworthiness of the data is lacked

*Following qualitative best practices, we have taken many steps to ensure the trustworthiness of our data during preparation, data collection, and data analysis. Our approaches align with strategies outlined in the following articles, and within the COREQ guidelines.*

- *JM Morse. Critical Analysis of Strategies for Determining Rigor in Qualitative Inquiry. Qualitative Health Research. 2015, Vol. 25(9) 1212–1222*
- *Cohen, DJ, and BF Crabtree.* ***Evaluative Criteria for Qualitative Research in Health Care: Controversies and Recommendations.*** *Annals of Family Medicine. 2008: 8(4); 331-339.*
- *Tong A, Sainsbury P, Craig J.* ***Consolidated criteria for reporting qualitative research (COREQ): a 32-item checklist for interviews and focus groups****. Int J Qual Health Care 2007; 19(6):349-357*

C. On the result and the discussion section

1. Not presented in logical and clear manner
2. The tables are not self-explanatory
3. Needs concise presentation
4. The discussion is relatively better but still needs more explanation with appropriate argument and refinement.

*We have revised the results section to improve clarity and added additional details to the discussion. In presenting the results, we followed the guidance outlined in the papers noted below. We have ensured all table titles are self- explanatory and updated content to ensure clarity.*

- *Sandelowski M. Writing a good read: strategies for re-presenting qualitative data. Res Nurs Health. 1998 Aug;21(4):375-82. doi: 10.1002/(sici)1098-240x(199808)21:4<375::aid-nur9>3.0.co;2-c. PMID: 9679813*
- *Sandelowski M, Leeman J. Writing usable qualitative health research findings. Qual Health Res. 2012 Oct;22(10):1404-13. doi: 10.1177/1049732312450368. Epub 2012 Jun 28. PMID: 22745362.*
- *Tong A, Sainsbury P, Craig J.* ***Consolidated criteria for reporting qualitative research (COREQ): a 32-item checklist for interviews and focus groups****. Int J Qual Health Care 2007; 19(6):349-357*

**Reviewer #3:**

1. language has major issues it needs to be revised

*Thank you for the feedback regarding the language in our manuscript. We understand the importance of clear and precise language in conveying our research. We have thoroughly reviewed the manuscript with this in mind and made additional edits for clarity.*

2. purpose and objectives needs revision

*Thank you for this comment. We have made edits to provide greater clarity, which also align with suggestions from reviewer #4*.

3. methodology needs more clarity and precision in writing it should separately discuss the setting, design, eligibility and then methods of collection of data

*We followed COREQ guidelines in the write-up of our manuscript and have made edits throughout the methods section to improve clarity.*

**Reviewer #4:**

Introduction section

1. Briefly explaining the UNAIDS 95-95-95 goals would provide all readers with context.

*We have included a brief explanation of these goals for context and it now reads: “Despite significant advances toward achieving UNAIDS 95-95-95 goals [1], whereby 95% of persons living with HIV should know their HIV status, 95% of persons diagnosed with HIV should receive antiretroviral therapy (ART), and 95% of all those receiving ART should have suppressed viral loads, youth living with HIV (YLH) remain at substantial risk of adverse outcomes.*”

2. Reordering the introduction to start with the specific challenges faced by Young People Living with HIV (YLH) could immediately engage the readers by addressing the main focus of the study. This could be followed by a discussion on the ATTACH study and its outcomes, and then the focus on sustainment and fidelity, creating a clearer narrative.

*We have organized our introduction to start with the magnitude of the problems faced by YLH and highlight the need for moving interventions from research to practice earlier on to emphasize the main goal of this work early in the introduction.*

3. Explicitly state what makes this study novel or unique. For example, mention if this is one of the first studies to evaluate post-trial sustainment and fidelity of an EBI for YLH in Kenya or similar contexts.

*We added this information to the strengths and limitations section and the sentence reads: “Evaluations to understand continued intervention use post-trial are not commonly conducted.”*

Method section

1. Using the Consolidated Framework for Implementation Research (CFIR 2.0) is appropriate and well-justified. However, a brief explanation of why CFIR 2.0 was chosen over other frameworks could enhance understanding

*We used the original CFIR framework in the initial trial and continued using it for consistency. CFIR was originally chosen to evaluate determinants of implementation across multiple levels of influence, including the intervention, the clinic setting, and contextual settings outside the clinic. We chose to apply the updated and newly available CFIR 2.0 framework for this study because it better reflects the relevant contextual factors in low—and middle-income settings when compared to the original CFIR. We have added this information to the methods section.*

2. It might also be beneficial to mention any specific training the interviewer received for this study to emphasize their preparedness further

*Thank you for your suggestion. We added this information to the method section and it now reads:*

*“Interviewers were all Kenyan women with substantial experience conducting qualitative interviews related to adolescent HIV and grounded in CFIR, and previous experience working on the ATTACH study. Prior to conducting the interviews, the senior qualitative researcher reviewed the interview guides with the interviewers to refine the interview tools and ensure comprehension.”*

3. The median interview duration of 31 minutes is noted, which provides a sense of the depth of the interviews. Including a range (e.g., minimum and maximum duration) could offer additional context on the variability of the interview lengths

*Thank you for your suggestion. We have included this information in the methods section and it now reads:*

*“Interviews were a median of 31 minutes (range: 16-66 minutes).*

4. Include examples of specific questions from the interview guide to illustrate how the interviews explored HCW experiences and implementation factors

*We have added the interview guide as supplemental material document 2.*

5. The flexibility in conducting interviews in English, Kiswahili, or Dholuo based on interviewee preference is commendable. It ensures that participants can express themselves comfortably and accurately. It would be helpful to elaborate on the translation process to ensure the accuracy and consistency of the transcriptions.
   Describe any quality control measures for transcription and translation to ensure data integrity

*We have added information regarding the translation and transcription process to the methods section and it now reads:*

*“All interviews were first transcribed verbatim and translated as needed by the qualitative interviewers, as they were most familiar with the conversation and context. Structured debrief reports were written by interviewers shortly after IDI completion to capture immediate feedback on interview content and quality.”*

6. The coding process is well-described, but it would be helpful to elaborate on how the team handled emergent themes that did not fit within the initial codebook.

*We have added the following sentence to the methods section to clarify how this was addressed:*

*“Emergent concepts not included in the CFIR-guided codebook were identified through open coding, discussed with the coding team in real time, and added to the codebook as deemed appropriate. When new codes were deemed necessary, previous transcripts were reviewed to apply the new codes.*”

Discussion Section

1. While workforce shortages and high staff turnover are mentioned, discuss specific strategies to mitigate these issues. For example, elaborate on how task shifting and peer champions can be systematically implemented.

*We have added this information to the discussion and updated the manuscript to incorporate the following relevant reference:*

*Rachlis B, Naanyu V, Wachira J, Genberg B, Koech B, Kamene R, et al. Community Perceptions of Community Health Workers (CHWs) and Their Roles in Management for HIV, Tuberculosis and Hypertension in Western Kenya. PLOS ONE. 2016 Feb 22;11(2):e0149412.*

*The text now reads:*

*“Workforce constraints, as we found in our study, are common challenges in health systems in many countries in sub-Saharan Africa [27–29]. Approaches to address this have previously included task shifting, task sharing, and aligning EBI delivery to fit within the existing workforce [30–32]. Examples of successful models include the incorporation of peer support to provide non-clinical educational or support services in clinics [33].*

2. The lack of integration into existing EMR systems has a negative impact on sustained implementation. However, the discussion could be strengthened by providing more details on why this integration was challenging and what steps could be taken to address this issue.

*Thank you for your comment. The EMR system is a national system, and multiple steps are involved in changing it and integrating new interventions. More stakeholder engagement would support this, which was beyond the scope of this study.*

*The discussion now reads: “ However, sustained implementation was negatively impacted by workforce shortages, high staff turnover, and lack of integration into existing EMR systems. Changing and integrating new interventions into the national EMR system requires multiple steps and increased stakeholder engagement, which was beyond the scope of this study.*”

3. The discussion references other studies evaluating factors influencing the sustainment of EBIs. It would be beneficial to provide a more detailed comparative analysis, highlighting how the findings of this study align with or differ from those of other studies.

*We have edited the discussion and it now reads:*

*“Our findings resonate with the broader context of addressing workforce constraints where strategies such as aligning evidence-based intervention (EBI) delivery with existing human resources are essential. Overall, studies on sustainment of EBIs show the importance of training, internal champions, and addressing workforce constraints in maintaining long-term use of interventions. Similar to other studies, our research emphasizes the role of supportive internal structures and champions in sustaining EBIs. Additionally, our study highlights the critical need for adaptable intervention models that align with existing workforce constraints.”*

4. Emphasize the impact of the findings on future research and practice. For instance, how can the identified determinants of sustainment be applied to other EBIs?

*Our analysis highlighted determinants of training, internal and external support, and intervention adaptability as key influences on sustainment. We have added a sentence in the conclusion section to relate these findings to sustainment of other EBIs and the text now reads:*

*“Our findings highlight key determinants of post-trial EBI sustainment, emphasizing the importance of training, internal and external support, and intervention adaptability. These factors should be considered for future intervention development. Strategies to ensure continued training and improve intervention integration into existing point-of-care systems can contribute to enhanced sustainability and scalability*.”

5. While the discussion focuses on sustainment one year post-trial, it would be valuable to consider the ATP's long-term sustainability. Are there any plans or recommendations for ensuring the continued use of the ATP beyond the initial post-trial period? Discussing potential long-term strategies and considerations could provide a more comprehensive view of sustainment.

*We agree that assessing long-term sustainability will be important. While this is beyond the scope of our current study, we have plans to evaluate long term sustainment in the future and have highlighted this in the strengths and limitations section, which now reads:*

*“Further studies are necessary to assess long-term sustainability in delivery beyond this initial timeframe.”*

6. The discussion could be enhanced by elaborating on the policy implications of the findings. How can the insights gained from this study inform policy decisions at the clinic, regional, or national levels?

*As stated in the response to comment #4, we have added to the conclusion that future EBI implementation may wish to consider the factors identified in our study to improve likelihood of sustainability. We also added the following sentence to the introduction section to clarify this point and the text now reads:*

*“After demonstrating effectiveness during the RCT, our team disseminated study results to the national HIV program and the ATP has now been included in revised adolescent HIV guidelines. We anticipate collaborating with the national program to scale the intervention.”*

**Reviewer #5:**

This is a fascinating qualitative study in the field of public health. It assesses the Sustainability of an evidence-based intervention supporting the transition to independent care for youth living with HIV in Kenya. The manuscript is well-written showing empirical evidence and meeting all standards of qualitative research.

1. However, the author should consider indicating the ethics approval number by the KNH/UoN in the methods section

*We have included the ethics approval number in the methods section.*
