# Supplementary material for: Sustainability of an evidence-based intervention supporting transition to independent care for youth living with HIV in Kenya
Source: PLOS Glob Public Health. 2025 Jan 13;5(1):e0004111. doi: 10.1371/journal.pgph.0004111 (PMC11981535; doi:10.1371/journal.pgph.0004111)
Supplement: Attachment — Submitted filename: Response To Reviewers_2.docx [file pgph.0004111.s004.docx]

**Reviewer #1**: The study background was presented logically and method was scientifically sound although, a qualitative study, the content and thematic analysis was sound.

*Thank you for this comment. We appreciate your review of this paper.*

**Reviewer #2**: Review Report

1. Title: Needs revision
2. Abstract: Lacks clarity
3. Background: Is relatively weak
4. Methods: Not explicit
5. Result and discussion: Lacks logical flow, brief and concise presentation

*Thank you for the review. We appreciate the reviewer’s perspective and would welcome further details on their comments, as this additional feedback would help us address the weaknesses they noted.*

**Reviewer #3**:

1. Consider breaking down long sentences into shorter, more direct ones can enhance clarity.

*We have reviewed the manuscript with this in mind and made edits accordingly*.

2. Improve the transition between the background on YLH challenges and the introduction of the ATTACH study. A brief connecting sentence could help maintain the flow of the narrative.

*Thank you for this comment. We’ve made edits to incorporate this feedback and it now reads:*

*“ Recognizing the need for structured support during this transition, the Adolescent Transition to Adult Care for adolescents living with HIV (ATTACH) study was conducted. This hybrid 1 implementation-effectiveness cluster randomized clinical trial (cRCT), which evaluated both the intervention effectiveness and factors influencing implementation simultaneously, developed and tested an Adolescent Transition Package (ATP) to support Kenyan YLH with transition from pediatric/adolescent focused care to adult care.”*

3. A brief statement on how the findings of this study might influence future interventions or public health strategies could add depth to the introduction.

*We have addressed this in the last paragraph of the discussion. We have also revised the introduction, which now reads:*

*“Using a qualitative approach to evaluate these outcomes provides a deeper understanding of HCW’s perspectives and experiences with ATP implementation, which can inform approaches to facilitate its scale-up and long-term integration into routine care in Kenya. Few studies in resource-limited settings address transition, making this program a potential model for other countries to replicate. Lessons learned from ATP delivery may also help inform scale-up and sustainability of other evidence-based interventions in Kenya and similar settings*.”

4. Addressing the challenges of high staff turnover by ensuring ongoing training and support for new staff is essential for the long-term success of ATP implementation.

*Thank you for this important comment. We agree with the reviewer and have highlighted this in the manuscript. We also made an edit to the discussion to emphasize this further and it now reads:*

“*However, training plans must accommodate existing staff turnover challenges by providing ongoing training and support for new staff and incorporate specific EBI training to sustain implementation*.”

5. Integrating ATP tools into existing electronic medical systems and aligning them with clinic workflows will improve documentation consistency and overall program fidelity.

*We agree with the reviewer and this is an important finding highlighted in our conclusion.*

6. The discussion could benefit from deeper exploration of the specific challenges faced, particularly regarding the integration of ATP into existing EMR systems. More details on how these integration issues specifically impacted the ATP’s use and sustainment would be valuable.

*We agree that this is an important next step in considering scale-up and sustainability. While this study did not focus on the specific details of integrating tools into the EMR systems, we have included relevant findings from the interviews and noted in the discussion that the process of changing the existing infrastructure was beyond the scope of the study. The EMR integration will be explored more in future work.*

7. While comparisons with other studies are made, the discussion could be strengthened by providing more detailed comparisons with similar programs or interventions in different settings.

*We have addressed this comment alongside comment #8 below.*

8. There could be a more detailed discussion on how the findings align or contrast with specific studies on similar interventions. This would provide a more nuanced understanding of where the ATP stands in relation to other EBIs.

*Thank you for the comments in numbers 7 and 8. Transitional programs are rare in this setting. We provided comparisons with other studies evaluating factors influencing the sustainment of EBIs. There are successful models that can provide examples of how similar interventions have been scaled and sustained, such as Kenya’s PrEP roll-out. We have added this example to the manuscript and it reads:*

*“As demonstrated by the PrEP roll-out in Kenya [38], government and clinic buy-in, integration of tools within existing national frameworks, and intervention alignment to clinic goals are critical for sustainment [39].”*

9. Recommendations for addressing the specific barriers identified (e.g., workforce shortages, high staff turnover, EMR integration) could be more detailed. Providing concrete strategies or examples from other programs that have successfully overcome these barriers would enhance the practical utility of the discussion.

*Examples of successful models are integrated and referenced throughout the discussion section and have also been addressed by edits made above (comments 4-8).*

10. The results section mentions that 30 HCWs participated in the study, but it does not address the justification for this sample size. Providing information on how this number was determined (e.g., power analysis, feasibility) would strengthen the validity of the findings.

*Thank you for this comment. Power analysis is not an appropriate approach in qualitative research. Rather, we used information power to guide our sampling, which is in line with best practices around saturation as outlined in the following reference:*

[Hennink, M. M., Kaiser, B. N., & Marconi, V. C. (2017). Code saturation versus meaning saturation: how many interviews are enough?. *Qualitative health research*, *27*(4), 591-608](https://www.ncbi.nlm.nih.gov/pmc/articles/PMC9359070/).

11. While gender and cadre distributions are provided, there is no discussion on how these distributions might influence the results. For instance, how might the predominance of female HCWs (73.3%) and the various cadres (e.g., peer mentors vs. clinical officers) impact the ATP’s implementation and feedback?

*Thank you for this question. The gender and cadre distributions reflect the staff distributions within HIV clinics in this setting. This information is provided to help interpret the results and assess applicability in different contexts. This study was not designed to analyze the impact of gender or cadre on ATP implementation*.

12. Although the training sources are mentioned, there is no further analysis of how the training by ATTACH study staff versus other HCWs might have impacted the implementation and effectiveness of the ATP. This could be an important factor in understanding variability in responses.

*The majority of participants (77%) were trained by ATTACH study staff. While the training source could have influenced the implementation, we have limited data to fully assess this impact*. *Training by non research staff will be better understood in future scale-up work.*

**Reviewer #4**: Thank you for the manuscript. This is a very important study for the region. The manuscript is generally well-written, but I have a few observations which, when addressed, will improve the manuscript. Most are minor issues to be explained or addressed.

1. Introduction
A. While the background is focused on Kenya, it could benefit from a broader perspective by briefly discussing how similar challenges and interventions are being addressed in other regions or countries. This would position the study within a global context and increase its relevance.

*We have addressed this based on comments from reviewer 3 and have made additions to the introduction so that it now reads:*

“*Using a qualitative approach to evaluate these outcomes provides a deeper understanding of HCW’s perspectives and experiences with ATP implementation, which can inform approaches to facilitate its scale-up and long-term integration into routine care in Kenya. Few studies in resource-limited settings address transition, making this program a potential model for other countries to replicate. Lessons learned from ATP delivery may also help inform scale-up and sustainability of other evidence-based interventions in Kenya and similar settings*.”

B. While the background mentions the lack of evidence on EBI sustainment post-trial, it would benefit from a brief discussion of why sustainment is particularly challenging in low-resource settings like Kenya.

*We have added the following sentence to the introduction to highlight the general challenges of sustainment in low resource settings:*

*“Given the double burden of disease, health care worker shortages and limited resources, sustainment of health interventions in low resource settings, including Kenya, remains particularly challenging [3].”*

C. The link between the challenges faced by YLH and the need for sustainment of EBIs could be made more explicit earlier in the section. This would help the reader immediately understand the importance of the study.

*Thank you for this comment. We have the following sentence in the first paragraph of the introduction:*

*“High HIV incidence coupled with poor clinical outcomes among YLH necessitates an urgent need for effective and sustainable evidence-based interventions (EBIs)*”

D. The section on the challenges associated with transitioning from pediatric to adult care is well-explained, but it could be enhanced by providing more specific examples of how these challenges manifest in practice. For instance, explaining how anxiety and reluctance to transition contribute to treatment interruptions could add depth to the discussion.

*Thank you for this comment. While these are really important questions, they were beyond the scope of this paper whose main focus is on delivery of transition tools. We have cited relevant literature on challenges youth face in the transition period and the importance of a structured transition approach.*

E. The background introduces the ATTACH study as a hybrid implementation-effectiveness cluster randomized clinical trial (cRCT). However, it would be helpful to briefly explain what a hybrid implementation-effectiveness trial is, as not all readers may be familiar with this study design. This could be done in a sentence or two.

*Thank you for this comment. We have added a brief explanation and it now reads:*

*“This hybrid 1 implementation-effectiveness cluster randomized clinical trial (cRCT), which evaluated both the intervention effectiveness and factors influencing implementation simultaneously, developed and tested an Adolescent Transition Package (ATP) to support Kenyan YLH with transition from pediatric/adolescent focused care to adult care*.”

2. Methods
A. While purposive sampling is appropriate for qualitative studies, it's essential to clearly articulate the specific criteria used for selecting participants. How were participants selected based on their roles, experience with ATP, and satisfaction scores?

*Thank you for this comment. Details on the selection criteria are included in lines 118-124. We have added examples of professional roles within the clinic to provide additional context and it now reads:*

“*Prior to IDI recruitment, all HCWs from intervention sites completed surveys to capture data on their experiences with ATP delivery (ex: How long have you been using the adolescent transition package tools), professional roles within the clinic (ex: peer mentor, counselor, clinician), and overall satisfaction with the ATP tools (ex: Are you satisfied with the way the Adolescent Transition Package is designed to be delivered). Survey responses were used to recruit a diverse sample of HCWs representing a range of cadres, experiences, and satisfaction scores*.*”*

B. Consider providing a justification for the sample size of two to three HCWs per clinic. Was this based on prior research, power calculations, or theoretical saturation?

*30 interviews provided sufficient information power to answer the research question. We followed guidelines outlined in this paper for our sampling approach and have ensured this citation is included in the the methods section as a reference.*

[Hennink MM, Kaiser BN, Marconi VC. Code Saturation Versus Meaning Saturation: How Many Interviews Are Enough? Qual Health Res. 2017 Mar;27(4):591–608.](https://www.ncbi.nlm.nih.gov/pmc/articles/PMC9359070/)

*We have also made an edit to the data collection section and it now reads:*

*“Using purposive sampling, we recruited two to three HCWs from each of the 10 ATTACH study intervention clinics to participate in semi-structured individual interviews (IDIs). Typically these clinics have a small number of staff, between 3-12 providers, who deliver a full range of services. Sampling of two to three HCWs per clinic allowed us to capture diverse perspectives across multiple clinics and ensure information power.”*

C. While the authors mention that the interview guide was informed by CFIR, it would be beneficial to provide more details about its content. What were the key themes explored in the interviews?

*To ensure clarity, we have ensured the interview guide is included as supplementary material accompanying the article. We have also revised the data collection section to provide additional details and it now reads*:

“*Interview guides were informed by all domains of the CFIR 2.0, and focused on exploring HCW experiences delivering the ATP and the factors supporting continued implementation post trial.*”

D. The authors mention using English, Kiswahili, and Dholuo. Were there specific criteria for language selection? How were potential language barriers addressed during the interviews and transcription process?

*All HCWs are fluent in English, but the spoken language in Kenya includes a mix of languages depending on the locality. We’ve made an edit to the data collection section and it now reads*:

*“Interviewers were all Kenyan women with substantial experience conducting qualitative interviews related to adolescent HIV and grounded in CFIR, previous experience working on the ATTACH study, and who are fluent in languages most commonly used in these settings. Prior to conducting the interviews, the senior qualitative researcher provided training on and reviewed the interview guides with the interviewers to refine the interview tools and ensure comprehension. Interviews were a median of 31 minutes (range 16- 66 minutes). They were audio recorded and conducted in a combination of English and Kiswahili or Dholuo, depending on interviewee preference.”*

E. The description of interviewer training is brief. What specific training did the interviewers receive in qualitative interviewing techniques, CFIR, and cultural sensitivity?

All the interviewers were Kenyan women with extensive engagement with the communities, previous experience working on the ATTACH study, and substantial experience conducting qualitative interviews. We’ve made edits to the data collection section to clarify this experience and it now reads:

“*Interviewers were all Kenyan women with substantial experience conducting qualitative interviews related to adolescent HIV and grounded in CFIR, previous experience working on the ATTACH study, and who are fluent in languages most commonly used in these settings. Prior to conducting the interviews, the senior qualitative researcher provided training on and reviewed the interview guides with the interviewers to refine the interview tools and ensure comprehension*.”

F. The authors mention a hybrid approach to codebook development. It would be helpful to elaborate on how the deductive and inductive coding processes were integrated. Were there specific guidelines for when to use each approach?

*Thank you for this comment. We have explained our process for inductive vs. deductive coding in the methods section lines 153- 158 and it reads:*

”*Deductively, predetermined codes based on the CFIR 2.0 were used to develop an initial codebook. The codebook incorporated a majority of CFIR constructs; however, several constructs within the process domain were found to be irrelevant for this analysis and were removed from the codebook prior to coding. Emergent concepts not included in the CFIR-guided codebook were identified through open coding, discussed with the team in real time, and added to the codebook as deemed appropriate*.”

*Our description of this process followed the guidelines of the following paper*:

[DeCuir-Gunby JT, Marshall PL, McCulloch AW. Developing and Using a Codebook for the Analysis of Interview Data: An Example from a Professional Development Research Project. Field Methods. 2011 May 1;23(2):136–55.](https://journals.sagepub.com/doi/10.1177/1525822X10388468)

G. The methods mention that some CFIR constructs were removed from the codebook. It would be helpful to specify which constructs were removed and why, as this could impact the interpretation of the results.

*As noted in the manuscript, the codebook incorporated most CFIR constructs; however, several constructs within the Process domain were deemed irrelevant for this specific analysis and were removed from the codebook prior to coding. These constructs were not applicable based on the data collected, and their exclusion was intended to ensure the codebook remained focused on the relevant constructs for our study.*

H. While the authors mention regular meetings to discuss codes and resolve discrepancies, it's important to report specific measures of inter-rater reliability (e.g., Cohen's kappa) to assess the consistency of coding.

*While inter-rater reliability measures can assess consistency of coding, there are some reservations to using a numerical approach to quantify agreement. We used alternative best practices to maintain rigor which are documented in lines 160-169 in the manuscript. The article below outlines these inter-rater reliability score reservations and additional standards for ensuring rigor in qualitative research*:

[O’Connor, C., & Joffe, H. (2020). Intercoder reliability in qualitative research: debates and practical guidelines. International journal of qualitative methods, 19, 1609406919899220](https://journals.sagepub.com/doi/10.1177/1609406919899220).

I. The description of reflexivity is brief. It would be beneficial to provide more details about how researchers addressed their personal biases and how these biases might have influenced the interpretation of data.

*We’ve made edits to the methods section to expand this description and it now reads:*

“*Members of the coding team engaged in ongoing reflexivity, including memoing and group discussions, throughout the entire project to consider their personal biases and discuss alternative interpretations of the data*.”

J. While the authors mention ethical approval and informed consent, it's important to address potential power dynamics between researchers and participants, particularly in a hierarchical setting like healthcare.

*Thank you for this comment. Since the participants were healthcare providers, the potential power dynamics were considered less concerning compared to scenarios involving more vulnerable groups, such as adolescents. However, we took steps to ensure participation was voluntary and confidential, minimizing any influence of hierarchical dynamics and encouraging honest and open feedback.*

K. The authors mention plans for a collaborative stakeholder workshop. It would be helpful to elaborate on how findings from this qualitative study will be shared with participants and other stakeholders to inform future interventions.

*Thank you for this important comment. We have presented preliminary findings to stakeholders at a two day dissemination workshop, which also focused on identifying next steps and priorities for future ATP implementation. This information is included in the accompanying inclusivity questionnaire.*

L. While there is a reference to inclusivity in global research, a summary or key points from the Supporting Information would provide context on how inclusivity was addressed in the study.

*The inclusivity questionnaire provides transparency on how inclusivity was addressed in our study, including community engagement, consent processes, and dissemination plans. The full details are included in the Supporting Information, and we have referenced this in the manuscript to guide readers to the relevant context*.

**Reviewer #5**: The manuscript has no dual publication, research, or publication ethics issues. Also, my earlier review issue about the ethical approval number has been addressed.

*Thank you for this comment. We appreciate your review of this paper.*
